# Transcriptome Profiling Reveals a Novel Mechanism of Antiviral Immunity Upon Sacbrood Virus Infection in Honey Bee Larvae (*Apis cerana*)

**DOI:** 10.3389/fmicb.2021.615893

**Published:** 2021-06-02

**Authors:** Yulong Guo, Zhengyi Zhang, Mingsheng Zhuang, Liuhao Wang, Kai Li, Jun Yao, Huipeng Yang, Jiaxing Huang, Yue Hao, Fan Ying, Hira Mannan, Jie Wu, Yanping Chen, Jilian Li

**Affiliations:** ^1^Key Laboratory of Pollinating Insect Biology of the Ministry of Agriculture, Institute of Apicultural Research, Chinese Academy of Agricultural Sciences, Beijing, China; ^2^Shanghai Suosheng Biotechnology Co., Ltd., Shanghai, China; ^3^College of Resources and Environmental Sciences, Henan Institute of Science and Technology, Xinxiang, China; ^4^Guizhou Provincial Animal and Poultry Genetic Resources Management Station, Guiyang, China; ^5^Department of Entomology, Faculty of Crop Protection, Sindh Agriculture University, Tando Jam, Pakistan; ^6^United States Department of Agriculture (USD) – Agricultural Research Service (ARS) Bee Research Laboratory, Beltsville, MD, United States

**Keywords:** *Apis cerana*, Chinese Sacbrood virus, transcriptome, antimicrobial peptides, Sirtuins

## Abstract

The honey bee is one of the most important pollinators in the agricultural system and is responsible for pollinating a third of all food we eat. Sacbrood virus (SBV) is a member of the virus family *Iflaviridae* and affects honey bee larvae and causes particularly devastating disease in the Asian honey bees, *Apis cerana*. Chinese Sacbrood virus (CSBV) is a geographic strain of SBV identified in China and has resulted in mass death of honey bees in China in recent years. However, the molecular mechanism underlying SBV infection in the Asian honey bee has remained unelucidated. In this present study, we employed high throughput next-generation sequencing technology to study the host transcriptional responses to CSBV infection in *A. cerana* larvae, and were able to identify genome-wide differentially expressed genes associated with the viral infection. Our study identified 2,534 differentially expressed genes (DEGs) involved in host innate immunity including Toll and immune deficiency (IMD) pathways, RNA interference (RNAi) pathway, endocytosis, etc. Notably, the expression of genes encoding antimicrobial peptides (*abaecin*, *apidaecin*, *hymenoptaecin*, and *defensin*) and core components of RNAi such as *Dicer-like* and *Ago2* were found to be significantly upregulated in CSBV infected larvae. Most importantly, the expression of Sirtuin target genes, a family of signaling proteins involved in metabolic regulation, apoptosis, and intracellular signaling was found to be changed, providing the first evidence of the involvement of Sirtuin signaling pathway in insects’ immune response to a virus infection. The results obtained from this study provide novel insights into the molecular mechanism and immune responses involved in CSBV infection, which in turn will contribute to the development of diagnostics and treatment for the diseases in honey bees.

## Introduction

The Asian honey bee *Apis cerana* is one of two honey bee species that have been truly domesticated and used in apiculture. It has adapted diverse environments, and its natural distribution is also broad, and is widely distributed in complex topographic regions with different habitats, diverse flora, and divergent climate in Asia (Hepburn and Radloff, 2011). Like its western counterpart, *Apis mellifera*, *A. cerana* also plays a vital role in agricultural production and biodiversity conservation. However, due to degradation of ecosystems, loss of biodiversity, overexploitation of natural resources, excessive use of pesticides, and introduction of exotic species, the original habitats of the Asian honey bee have shrunk by 75% over the past century and the populations of managed Asian honey bees have declined by 80% (He and Liu, 2011; Chen et al., 2017). As a result, in 2006, *A. cerana* was listed as an endangered species.

*A. cerana* suffers from a variety of diseases caused by viruses, bacteria, fungi, and parasites. Of all the pathogens causing diseases in honey bees, Sacbrood virus (SBV) is the most dangerous pathogen of *A. cerana*. SBV belongs to *Iflaviridae*, a viral family of positive-sense single-stranded RNA viruses infecting insects (Chen and Siede, 2007). While SBV infects both brood and adult stages of honey bees, the larval stage is the most susceptible to SBV infection. Infected larvae fail to pupate while ecdysial fluid rich in SBV accumulates beneath the unshed larval cuticle, forming the sac, hence the name “Sacbrood.” Sacbrood disease was first described in the western honey bee *A. mellifera* in 1913 and later in *A. cerana* in 1972 (Chen et al., 2017). Since then, the catastrophic outbreaks of the SBV disease have occurred periodically in a cycle of every 6–7 years, causing massive deaths and collapse of entire colonies in Asia. According to historical records, SBV disease killed 100% of *A. cerana* colonies in Thailand in 1976, 95% of *A. cerana* colonies in India in 1978, greater than 90% of *A. cerana* colonies in China and completely destroyed the Korean apiculture industry in 2010 (Bailey et al., 1982; Verma et al., 1990; Choe et al., 2012). The severe losses of *A. cerana* populations across Asia due to SBV disease were caused by a variety of strains of SBV reflecting their geographic isolations, namely Thai Sacbrood virus, Chinese Sacbrood virus, Korean Sacbrood virus, etc. (Bailey et al., 1982; Choe et al., 2012).

Chinese Sacbrood virus (CSBV) is a geographic strain of SBV isolated from the Asian honey bee *Apis cerana* in China and has been catastrophic for the Chinese beekeeping industry (Liu et al., 2017). Nonetheless, the molecular mechanisms underlying SBV disease pathogenesis and host susceptibility to the viral infection remain poorly understood. The advent of deep sequencing technologies has revolutionized the biological sciences and enable the measurement of unbiased, large-scale, genome-wide gene expression patterns. In order to have a better understanding of host responses to CSBV infections, we employed an RNA-Seq approach (Wang et al., 2009; Marguerat and Bähler, 2010; Mutz et al., 2013) to unravel global host transcriptional changes in CSBV-infected larvae. Furthermore, we conducted RT-qPCR to validate the differential expression of selected differentially expressed genes (DEGs). The knowledge and information gained from this study will provide novel insights into mechanisms about how host antiviral immune responses are generated during the course of a natural infection by CSBV, thereby leading to the development of effective disease management strategies.

## Materials and Methods

### Samples Collection and CSBV Identification

CSBV infected 4-instar larvae with recognized SBV disease symptoms were collected from three *A. cerana* colonies maintained in the apiary of the Institute of Apicultural Research, Chinese Academy of Agricultural Sciences (CAAS) for transcriptome analysis and qRT-PCR. The three adjacent healthy colonies of 4-instar larvae that were identified to be negative for CSBV infection were used for sampling healthy larvae as a negative control. Individually collected larvae were treated with 75% alcohol and subjected to RNA extraction using the QIAGEN RNeasy Mini Kit following the manufacturer’s instructions. The concentration of extracted RNA was measured by using a Nanodrop2000. The quality of RNA was confirmed using 1% TAE (Tris-acetate-EDTA) agarose gel electrophoresis. cDNA was synthesized from RNA using random hexamer primers and reverse transcriptase with the QIAGEN Reverse Transcription Kit following the manufacturer’s instructions. PCR assay was performed for each cDNA sample to confirm the status of CSBV infection with a pair of CSBV primers (Forward: 5′-GACCCGTTTTCTTGTGAGTTTTAG-3′; Reverse: 5′-GTGTAGCGTCCCCCTGAATAGAT-3′) (Ma et al., 2011). The specificity of the PCR product was visualized on 1% agarose gel electrophoresis, sequenced, and analyzed using the BLAST server at the National Center for Biotechnology Information, NIH.

### Host RNA-Sequencing

The Illumina Hiseq sequencing platform was used for transcriptome sequencing of ten CSBV infected larvae and nine health larvae. An Illumina PE library was constructed for 2 × 150 bp sequencing, and the obtained sequencing data were subject to quality control. The full-length cDNA was fragmented and ligated to an Illumina paired-end adapter for PCR enrichment and sequencing. The Illumina reads were processed to remove low-quality sequences, adaptor and to eliminate sequences of rRNA and tRNA.

### RNA-Seq Data Analysis

The information about the methods used to analyze the transcriptomic data is as follow: first, SeqPrep software was used to trim the raw data. (a) removed the adaptor sequence in reads, and removed the reads that have not been inserted due to the adaptor self-connection; (b) the base with low quality (quality value less than 20) at the end (3′ end) of the sequence was pruned away; (c) removed reads with N ratio over 10%; (d) discarded the adapter and any sequence whose length was less than 20 bp after quality pruning. SeqPrep softwares^1^ were used to deal with quality trimming. Secondly, Mapping reads to reference genome of honeybee (*Apis cerana*) and analysis profile of gene expression. The TopHat2 (Trapnell et al., 2009) software was used to map reads to reference genome of *Apis cerana* (version: ACSNU-2.0). RSEM software (Li and Dewey, 2011) was used to calculate gene expression level, and FPKM (Fragments Per Kilobase of Transcript per Million Fragments Mapped) (Mortazavi et al., 2008) was used as criteria of measuring gene expression.

For differential expression analysis, the edgeR (Robinson et al., 2010) was used to conduct differential gene expression analysis. Differential expression calculation was based on gene read count and a negative binomial distribution model. In this case, the screening criteria of significantly differentially expressed genes was FDR < 0.05 and | log2FC| ≥ = 1. Clustering analysis was conducted to gain expression patterns of Differentially Expressed Genes (DEGs). The distance algorithm was adopted (Spearman correlation between samples, Pearson correlation between genes, and the complete algorithm were adopted in the distance method).

For functional analysis of differentially expressed genes, Goatools (Klopfenstein et al., 2018) was used to perform Gene Ontology (GO) enrichment analysis based on Fisher’s exact test. The genome of *A. cerana* was used as the background to determine GO terms enriched in the DEG dataset using the hypergeometric test. FDR, as a corrected *P*-value, was used to control the false positive rate, FDR (<0.05) as a threshold to identify significantly enriched terms. DEGs were classified into three categories; biological process (BP), cellular components (CC), and molecular function (MF). Then, the same enrichment method was used to conduct KEGG pathway enrichment analysis. KOBAS (Xie et al., 2011) was performed to identify significantly enriched Kyoto Encyclopedia of Genes and Genomes (KEGG) pathways in the DEG datasets based on Fisher’s exact test and a corrected *P*-value (FDR ≤ 0.05) as a threshold. Next, Qiagen’s Ingenuity Pathway Analysis system (IPA^2^) was used to analyze the significant differentially expressed genes (DEGs, FDR ≤ 0.05). Briefly, the DEGs were first converted to their *Drosophila melanogaster* homologs based on BLAST (BLAST alignment parameter: e-value of 1e–5), then uploaded into the IPA system for core analysis and overlaid with the built-in knowledge base.

### Quantitative PCR for Quantification of Candidate DEGs

The expression levels of selected DEGs were confirmed by quantitative PCR (qPCR) in individual CSBV-infected bees from three colonies (10 bees/colony). Healthy worker larvae (10 bees/colony) were collected from three colonies as a control. The primer pairs evaluated in the study are included in Supplementary Table 9. Total RNA extracted from each bee samples was used in cDNA synthesis for downstream qPCR. qPCR was performed in a total reaction volume of 20 μL containing the following reagents: 10 μL SYBR Green PCR Master Mix (2×), 2 μL QN ROX Reference Dye, 0.8 μL of each primer (10 μmol/L), 4.4 μL ddH_2_O, 2 μL cDNA. The qPCR reactions were performed on a MX3000P system (Axygen) using the following cycle conditions: 95°C for 3 min, 35 cycles of 95°C for 30 s, 60°C for 30 s, and 72°C for 30 s. A melting curve was observed at the end of each run to confirm the specificity of each primer. Each sample was carried out in triplicate. Each cDNA sample was normalized by mRNA level of a housekeeping gene beta-actin. The relative expression levels of the selected DEGs were interpreted by comparative Ct method (2^–ΔΔ^ ^Ct)^ (Livak and Schmittgen, 2001).

## Results

### Sequencing Data Quality Control and Detection of Chinese Sacbrood Virus in Two Groups

**FIGURE 1:**
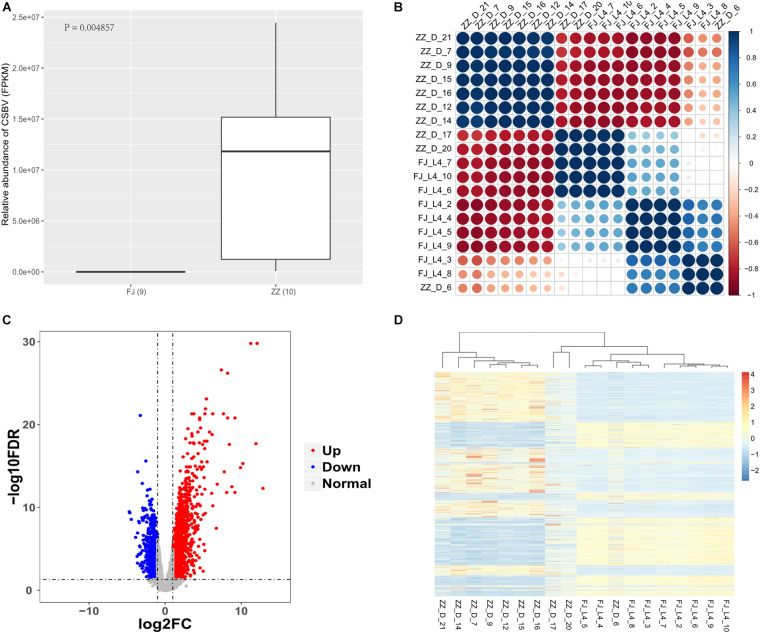
Analysis of virus content and gene expression pattern in samples. **(A)** The FPKM of Sacbrood Virus between the infected group (ZZ) and the healthy group (FJ) (*P* = 0.004857). **(B)** The correlationship of the samples. The correlation coefficient is more closer to absolute value of one, the similarity is higher among samples. The samples with bigger size, and darker color have more stronger correlationship to each other. The correlationship among the samples is calculated by the FPKM. **(C)** The volcano plot of the differentially expressed genes. The red dot is an upregulated gene, and the blue dot is a downregulated gene. The gray dot is a gene which has no significantly differentially expressed gene. The x-axis is the log of the fold change of FPKM which is between the treatment group and the control group. The y-axis is the negative log of the FDR. The FPKM is to standardize the relative abundance of expression of genes. The screening criteria for significantly differentially expressed genes are FDR < 0.05, and | log2(Fold change)| > 1. **(D)** The expression profile of the differentially expressed genes. The red color is upregulated, and the blue color is downregulated. The FPKM is to standardize the relative abundance of the gene expression. The z-score value with FPKM is used to draw the heatmap. The genes with *P* < 0.05 were used to cluster here.

The quality control results of the host sequencing data showed that the Q30 percentage value of the 19 samples was at an average of 94%, and the base error rate was at an average of 1%. After filtering the host RNA-Seq reads, the clean reads of the control samples were at an average of 1.1% and the clean reads of the treatment group were at an average of 52%. The relative abundance of CSBV reads in the CSBV-infected group was up to about 97.6% on average, while that of the healthy group was zero (Figure 1A and Supplementary Tables 1, 2).

### RNA-Seq Analysis of the Host Transcriptome

#### The Analysis of Correlationship of All the Nineteen Samples

We used RNA-seq data to analysis the correlationship of all the nineteen samples. The nine samples of healthy group were similar expression pattern. The seven tenth of the infected-CSBV group were the similar expression pattern, the other three samples were much more like that of the healthy group (Figure 1B and Supplementary Table 3).

#### Differentially Expressed Genes (DEGs) Function Analysis

**FIGURE 2:**
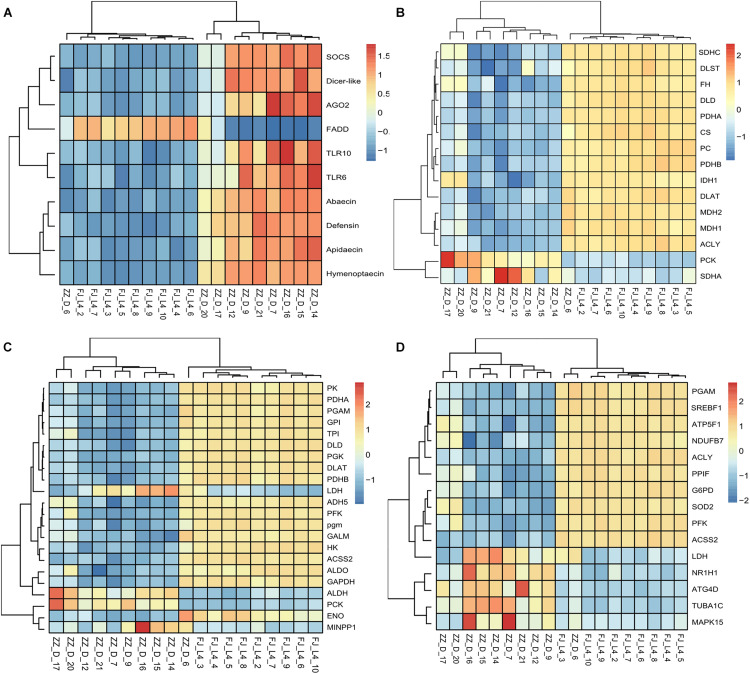
Heatmaps of candidate differentially expressed genes (DEGs). The function “aheatmap” in the R package named “NMF” were used to analyze the clustering. The distance measure used in clustering rows and columns was “euclidean,” and the clustering method used to cluster rows and columns was hclust with “complete.” **(A)** The expression pattern of the differentially expressed genes including antimicrobial peptides, RNA interference, and Toll like receptor gene expression. **(B)** The expression pattern of the differentially expressed genes involved in the tricarboxylic acid (TCA) cycle of energy metabolism. The rate-limiting enzyme including *DLD*, *CS*, *DLST*, and *IDH1* were down-regulated in the infected group. **(C)** The expression pattern of the differentially expressed genes involved in the Glycolysis. The rate-limiting enzyme including *HK*, *PFK*, and *PK* were down-regulated in the infected group. **(D)** The expression pattern of the differentially expressed genes involved in the Sirtuin pathway. The genes whose RNAseq data were consistent with the qPCR verification data were *ACLY*, *ATP5F1B*, *G6PD*, and *PFK*. These genes are also closely involved in energy metabolism.

**FIGURE 3:**
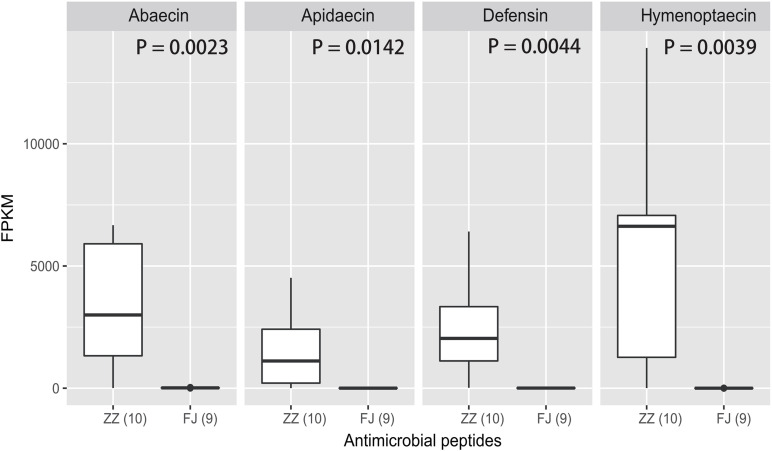
The expression levels of the four AMPs in the treatment and the control. The four antimicrobial peptides are *abaecin*, *apidaecin*, *defensin*, and *hymenoptaecin*. FPKM is a criteria to represent the relative abundance of the four genes. The analysis of significant difference was conducted by *t*-test (abaecin: *P* = 0.0023; apidaecin: *P* = 0.0142; defensin: *P* = 0.0044; hymenoptaecin: *P* = 0.0039).

**FIGURE 4:**
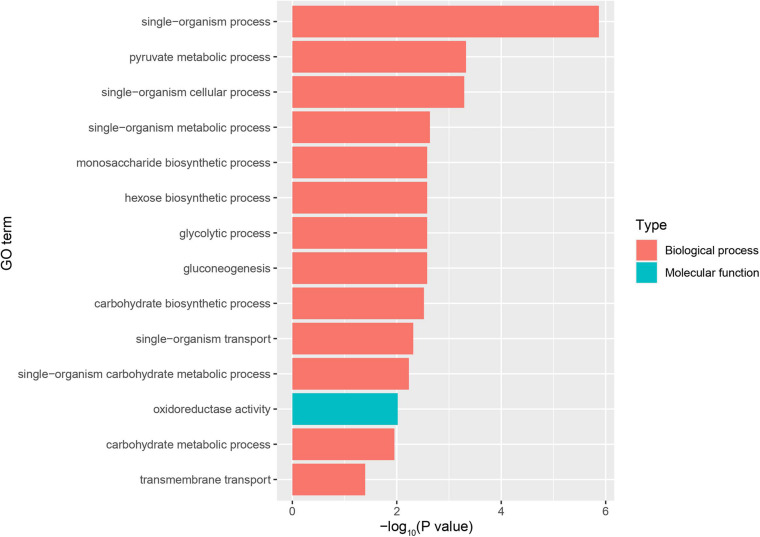
GO enrichment plot of differentially expressed genes (DEGs) between the infected group and the healthy group. The x-axis is the negative logarithm of the *p*-value, and the y-axis is Goterm. Go Term shows entries for Top14 (*P* bonferroni <0.05).

**FIGURE 5:**
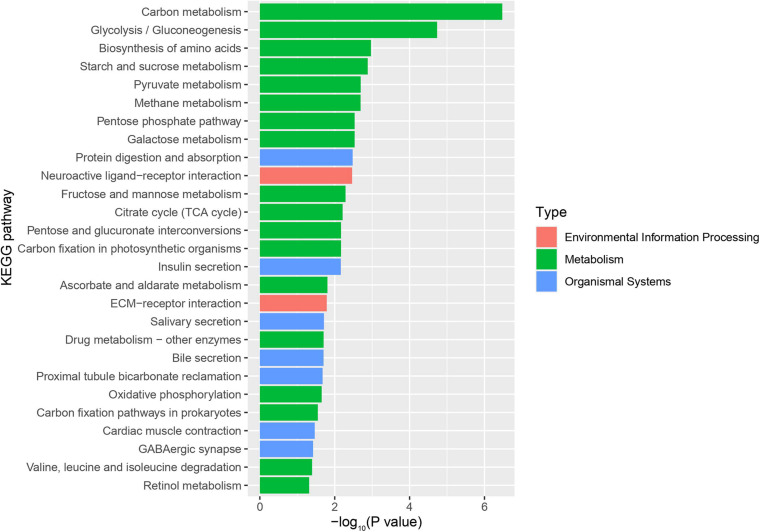
KEGG pathway enrichment plot of differentially expressed genes (DEGs) between the infected group and the healthy group. The x-axis is the negative logarithm of the *p*-value, and the y-axis is KEGG pathway (*P*-value < 0.05).

Using RNA-Seq to obtain genome-wide expression profiles of the DEGs in the larval bodies of the CSBV-infected larval group and the healthy larval group, we identified 2,534 genes that were significantly differentially expressed (FDR < 0.05; Supplementary Table 4). In the CSBV-infected group, 1,689 genes were upregulated and 845 genes were downregulated (Figure 1C). The DEGs of the CSBV-infected group and the healthy group had distinct expression profiles and were clearly separated according to hierarchical clustering analysis. In fact, most DEGs between the CSBV-infected group and the healthy group showed opposite expressed patterns; i.e., the DEGs were downregulated in the healthy group while are upregulated in the CSBV-infected group (Figure 1D). Specifically, the genes of antimicrobial peptides (AMPs) including *abaecin*, *hymenoptaecin*, *apidaecin*, and *defensin* had significantly higher levels of expression in the CSBV-infected group, compared to the healthy group. All four AMPs were significantly different within the two groups (*P* < 0.05; Figures 2A, 3). Gene ontology analysis revealed that the DEGs were significantly enriched in metabolic pathways including pyruvate metabolic process, glycolytic process, gluconeogenesis, carbohydrate metabolic process, oxidoreductase activity, and transmembrane transport (Figure 4 and Supplementary Table 5). The DEGs enriched in the KEGG pathways included carbohydrate metabolism, glycolysis, biosynthesis of amino acids, starch and sucrose metabolism, pyruvate metabolism, galactose metabolism, the pentose phosphate pathway, and neuroactive ligand-receptor interaction (Figure 5 and Supplementary Table 6).

**FIGURE 6:**
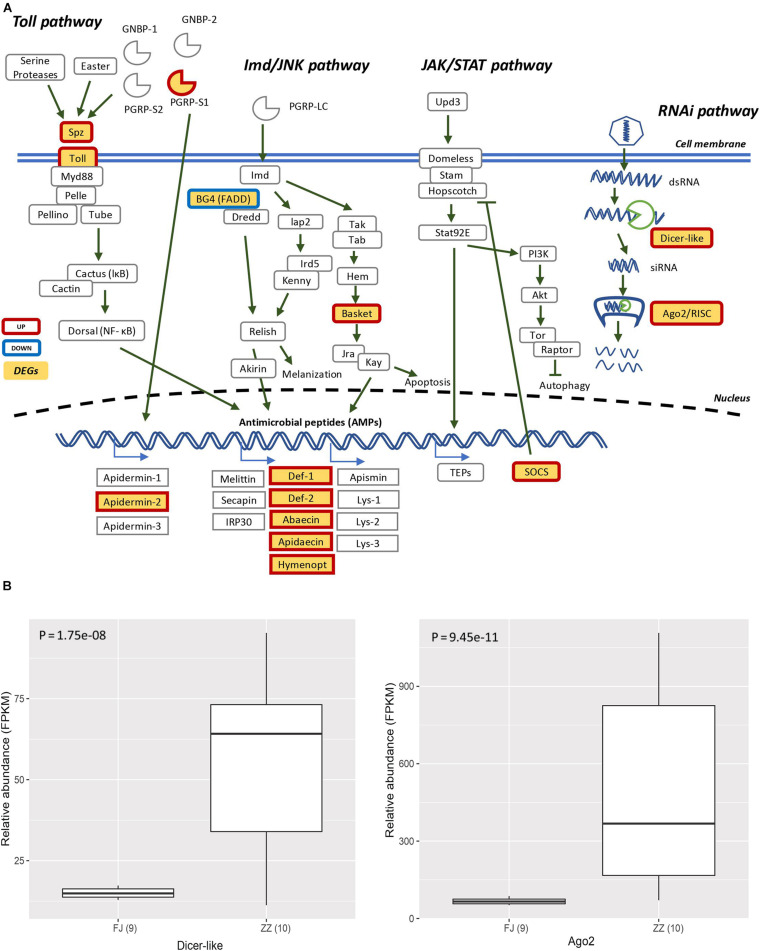
**(A)** The Signaling pathway of the canonical innate immune response of the honey bee. The pathways are those including Toll pathway, Imd/JNK pathway, JAK/STAT pathway, and RNAi pathway. The genes filled with yellow are differentially expressed genes (DEGs). The genes with red border are upregulated, and the genes with blue border are downregulated. **(B)** The DEGs (Dicer-like and Ago2) were involved in RNA interference (Dicer-like: *P* = 1.75e–08; Ago2: *P* = 9.45e–11).

The DEGs involved in antiviral response-associated signaling pathways almost were significant up-regulated. The gene Toll which encode Toll like receptor was up-regulated. The gene Basket involved in Imd pathway indirectly induced the production of antimicrobial peptides and apoptosis. Two key components of the RNAi pathway, *Argonaute-2* (*Ago2*) and *Dicer-like* were also up-regulated (Figures 6A,B).

In particular, the expression of genes involved in carbohydrate metabolism and energy metabolism such as the citrate cycle, glycolysis, the pentose phosphate pathway and oxidative phosphorylation was significantly altered in CSBV-infected bees. Further, the results also showed that genes encoding citrate synthase (CS), isocitrate dehydrogenase (IDH1), dihydrolipoyl dehydrogenase (DLD, and dihydrolipoyl transsuccinylase (DLST) which are enriched in the TCA cycle, an important aerobic pathway for the final steps of the oxidation of carbohydrates and fatty acids, were significantly downregulated. In the process of glycolysis, all three rate-limiting enzymes, hexokinase encoded by HK, phosphofructokinase encoded by pfkA, and pyruvate kinase encoded by PK, were also downregulated here. Some subunits of NADH dehydrogenase, succinate dehydrogenase, cytochrome c oxidase, F-type ATPase, and V-type ATPase of the oxidative phosphorylation were also downregulated (Figures 2B,C and Supplementary Table 7).

Most importantly, we found that 15 DEGs were associated with the Sirtuin signaling pathway (Figure 2D and Supplementary Table 8). For example, *ATG4D* is necessary for autophagy and regulated by *Sirt1.* Both *ACLY* and *ACSS2* participated in the synthesis of Acetyl-CoA. *ACLY*, was an enzyme of the TCA cycle in the cytoplasm, while *ACSS2* was in the mitochondria. *SOD2* was involved in oxidative stress and ROS detoxification. The other three DEGs, *PGAM*, *G6PD*, and *ATP5F1B*, were involved in oxidative phosphorylation, the pentose phosphate pathway, and ROS accumulation. The other DEGs were involved in pathways of adipogenesis and apoptosis (Figure 8).

To validate the expression level of the DEGs in the Sirtuin signaling pathway, nine DEGs were selected to perform quantitative PCR (qPCR) assays. Four genes including *ACLY*, *PFKM*, *G6PD*, and *ATP5F1B*, were found to be significantly downregulated (*P* = 1.806e–07; *P* = 1.129e–05; *P* = 2.703e–08; *P* = 1.53E–05) in CSBV-infected bees. The relative quantitative PCR results of the four genes (*P* = 1.1e–04; *P* = 7.834e–06; *P* = 2.366e–06; *P* = 2.642e–08) were consistent with the expression pattern shown in the RNA-Seq data (Figure 8).

## Discussion

**FIGURE 7:**
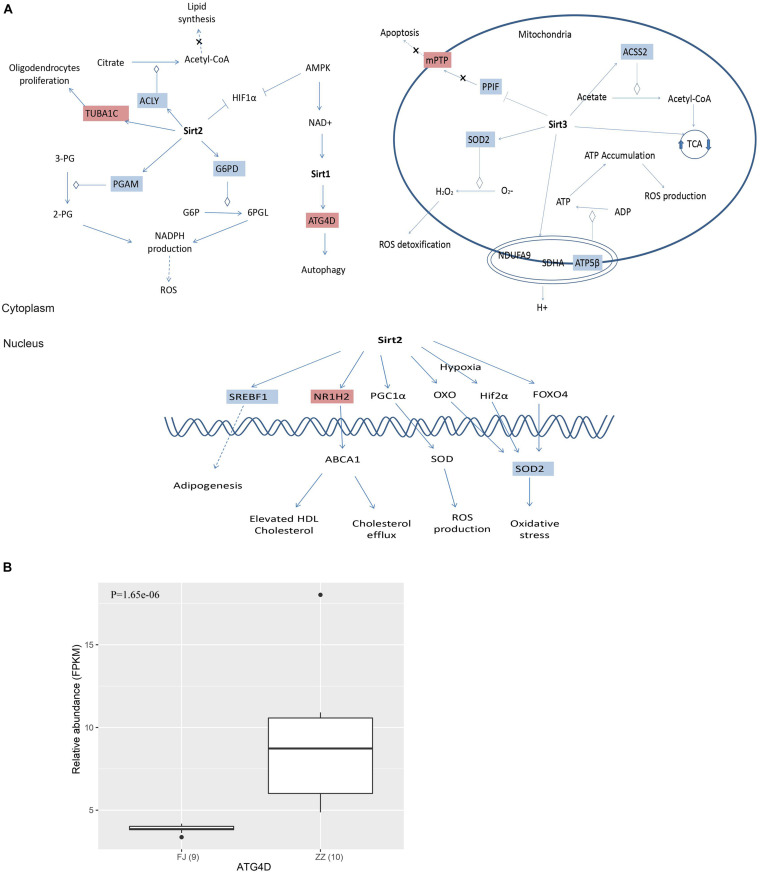
**(A)** The differentially expressed genes (DEGs) were invovled in Sirtuin signaling pathway. Sirtuins are class III histone deacetylase enzymes that use NAD^+^ as a co-substrate for their enzymatic activities. There are 15 DEGs involved in the Sirtuin pathway, and the DEGs are associated with energy metabolism, oxidative stress and apoptosis. **(B)** *ATG4D* associated with autophagy was significant differential expressed between the infected group and the healthy group.

The association of CSBV with high mortality of Asian honey bee colonies has led to an increased awareness of the risks of viral infections on bee health. It is critically important to understand which host genes were repressed or activated in response to the virus infection, thereby identifying prognostic biomarkers and drug targets for the early diagnosis and treatment of the disease. There are two key findings of the present research. First, the functional analysis of the DEGs revealed that the expression of genes involving immune defense and energy metabolism was significantly altered in response to the CSBV infection (Figure 2), reflecting intrinsic connection between metabolism and innate immune system (Ayres, 2020). Second, regulation of Sirtuin genes expression provides the first evidence of the involvement of Sirtuin signaling pathway in honey bees’ response to CSBV infection (Figure 7). This study represents the first comprehensive analysis of host changes on a global scale upon CSBV infection and the information obtained from this study will contribute significantly to the rational design of drugs for the treatment of honey bee viral diseases.

**FIGURE 8:**
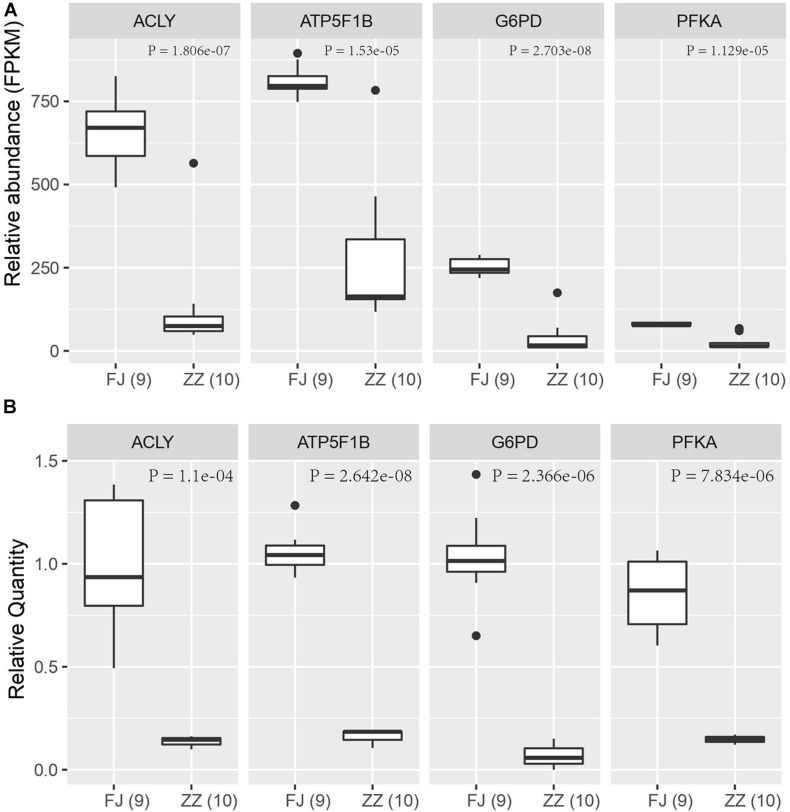
The qPCR validation of four differentially expressed genes in the Sirtuin pathway. **(A)** The relative abundance of four DEGs based on FPKM in RNA-Seq data. **(B)** The relative quantity (RQ) of four DEGs by qPCR assay. The significant difference of the infected group (ZZ) and the healthy group (FJ) was analyzed by *t*-test with *P* < 0.05.

The innate immunity represents insects’ first line of defense against invasion of infectious agents and is composed of cellular and humoral responses (Hoffmann, 1995). Humoral immune response is characterized by the synthesis and secretion of antimicrobial peptides (AMPs), small cationic peptides that penetrate microbial membranes and kill pathogens directly (Zasloff, 2002). The rapid production of AMPs in response to disease infection is also an integral part of honey bee humoral immunity. The expression of genes encoding AMPs in honey bees are regulated by intracellular signaling pathways Toll and Imd/JNK and could be induced by infection of bacteria, fungi, and viruses (Evans et al., 2006; Nazzi et al., 2012; Flenniken and Andino, 2013; Chen et al., 2014; Kuster et al., 2014). A previous study reported that expression of AMPs was upregulated in adult honey bees after injections of Escherichia coli, saline buffer, bee pathogen Paenibacillus larvae, or wounding (Evans et al., 2006). Our transcriptome profiling revealed many DEGs associated with honey bees’ immune responses to CSBV infection (Figure 4 and Supplementary Table 4). In accordance with previous reports, the expression of well-known described AMPs including apidaecin, hymenoptera, abaecin and defensin was found to be upregulated in CSBV infected larvae (Figure 3), this suggusts that AMP genes were induced not only by bacteria and fungi but also by some kinds of viruses like CSBV, DWV, and IAPV. Honey bee antiviral responses include not only immune pathways but also RNA interference (RNAi) (Mukherjee and Hanley, 2010; Kingsolver et al., 2013; Merkling and van Rij, 2013). Deformed wing virus (DWV) is the most common virus found in honey bee colonies. The immune pathways mounting a response against DWV infection in honey bees include RNAi, Toll, Imd, autophagy, and endocytosis (Nazzi et al., 2012; Chen et al., 2014; Kuster et al., 2014; Ryabov et al., 2014). In our study, the same immune signaling pathways were found to be triggered by CSBV infection. In addition to the upregulation of AMP genes controlled by the Toll and Imd pathways as well as genes involved autophagy and oxidative phosphorylation-related genes (Supplementary Table 7). Our study also showed that the expression of two core components of the RNAi pathway, *Dicer-like* and *Argonaute-2* (*Ago2*), was upregulated in CSBV-infected larvae (Figure 2A). RNAi-mediated antiviral defense has been identified and described in honey bees (Niu et al., 2014; Brutscher and Flenniken, 2015). When honey bees were experimentally infected with Israeli acute paralysis virus (IAPV), the levels of *Ago-2* and *Dicer-like* expression were markedly upregulated as compared to negative control of uninfected bees (Galbraith et al., 2015). The upregulation of *Dicer-like* and *Ago2* expression in CSBV infected larvae observed in our study suggests that RNAi machinery was triggered by the CSBV infection and presumably exerted its function to cleave the viral RNA through the action of Dicer-like and Argonaute (AGO) proteins. This result provides evidence that that RNAi pathway has been implicated in honey bees’ antiviral response to CSBV infection, and suggests that RNAi is a general antiviral response mechanism in virus infection.

Viruses depend entirely on their hosts’ cellular metabolism as energy resources to support their replication (Sanchez and Lagunoff, 2015). As a result, virus infections can dramatically alter host metabolic pathways and in turn the infection-induced alteration in metabolism can influence hosts’ immune functions to viral infections. Our results of RNA-seq and qPCR analyses showed that the DEGs including *ACLY*, *ACSS2*, *ATP5F1B*, *SOD2*, *PGAM*, *G6PD*, ATP citrate lyase that were associated with the tricarboxylic acid (TCA) cycle and energy metabolism were downregulated in the CSBV-infected bees. Concurrently, a large number of genes encoding NADH dehydrogenase, succinate dehydrogenase, cytochrome bc1 complex, cytochrome c oxidase, and ATP synthase that are involved in and oxidative phosphorylation and fatty acid oxidation were downregulated. Also, genes encoding phosphofructokinase (*PFKA*) which is one of the most important regulatory enzymes in glycolysis and G6PD, glucose-6-phosphate dehydrogenase involved in the pentose phosphate pathway (PPT) were found to be down-regulated in CSBV infected larvae (Figures 8A,B and Supplementary Tables 7, 8). These interconnected metabolic pathways including TCA cycle, glycolysis, the pentose phosphate pathway (PPP), oxidative phosphorylation, and the fatty acid oxidation were reported to have a role in the immune responses to various infectious diseases on both the cellular and the organismal levels (Ayres, 2020). The down-regulation of genes associated with diverse functions of cellular metabolic pathways in our study indicated that energy metabolism/biogenesis that are required for proper immune functions was seriously altered due to CSBV infection.

Sirtuins are a highly conserved family of proteins, these protein activity can prolong the lifespan of model organisms such as yeast, worms and flies (Verdin et al., 2010). Sirtuins are class III histone deacetylases that use NAD^+^ as a co-substrate for their enzymatic activities. In mammals, there are seven Sirtuin members (SIRT1-7) that are present in different cellular compartments with SIRT1, SIRT6, and SIRT7 predominantly localize to the nucleus, SIRT2 cytoplasmically located; and SIRT3, SIRT4, and SIRT5 being mitochondrial (Vassilopoulos et al., 2011). The mitochondrial sirtuins function in energy production, metabolism, apoptosis and intracellular signaling (Verdin et al., 2010). There are multiple enzymatic activities associated with SIRTs. In addition to their deacetylase activity, SIRTs are involved in other enzymatic activities including ADP ribosylation (SIRT1, SIRT4, and SIRT6), desuccinylation and demalonylation (SIRT5), delipoylation (SIRT4), and demyristoylation and depalmitoylation (SIRT6) (Koyuncu et al., 2014; Budayeva et al., 2016). The core molecular machinery of autophagy which named the “autophagy proteins” orchestrates diverse aspects of cellular responses to other dangerous stimuli such as infection, and autophagy pathway and proteins play a crucial role in immunity and inflammation (Levine et al., 2011). A study reported that SIRT1 has been associated with the induction of autophagy and the regulation of inflammatory mediators (Owczarczyk et al., 2015). A more recent study shows that SIRT1 regulates mitochondrial function and immune homeostasis in respiratory syncytial virus infected dendritic cells (Elesela et al., 2020). SIRT2, a nicotinamide adenine dinucleotide (NAD^+^)-dependent class III histone deacetylase, was found to be upregulated in wild-type hepatitis B virus (HBV WT)-replicating cells, leading to tubulin deacetylation (Piracha et al., 2018). SIRT3, SIRT4, and SIRT5 are mitochondrial deacetylases regulating a wide range of metabolic pathways that are known to be altered during viral infection as confirmed in our study (Koyuncu et al., 2014; Betsinger and Cristea, 2019). Of fifteen DEGs associated with and regulated by Sirtuin signaling pathway in our study (Supplementary Table 8), *ATG4D* (Autophagy Related 4D Cysteine Peptidase), a regulator of autophagy which is a mechanism that protect cells from degradation under stress conditions such as energy deprivation, and virus infection, was significantly upregulated after CSBV infection. Meanwhile, genes encoding the subunits of dehydrogenase, cytochrome c oxidase, ATPase of the oxidative phosphorylation as well as *PFKA* (phosphofructokinase) of glycolysis were down regulated in CSBV infected larvae. it is conceivable that down-regulation of metabolic pathways which are mediated by SIRTs would lead to insufficient fuel supply and immune suppression in infected bees.

In sum, this paper provides valuable insights into honey bee transcriptome responses to CSBV infection. Given the significant finding that CSBV-infected larvae displayed a marked upregulation of many genes involved in Sirtuin signaling pathway, we speculate that sirtuin inhibitors could be potently antiviral agents against CSBV infection in honey bees.

## Conclusion

Sacbrood virus have negative influence to honey bee larvae. The interaction between the host and the virus is complex. We showed that a large number of the genes had greatly changed in transcriptional regulation. Many genes which have relative to energy metabolism had down regulated. Other differentially expressed genes had play an important roles in immunity, oxidative stress, autophagy, and apoptosis. Interestingly, these important metabolism process is involved in a novel regulated pathway named sirtuin pathway. Our results showed that Sacbrood virus infection lead to activation of immunity system and dysfunction of energy metabolism involved in respiratory chain. These genes may be as targets of antiviral therapy to provide a new strategy for honey bee virus infection.

## Data Availability Statement

The raw sequence data reported in this paper have been deposited in the Genome Sequence Archive (88) in BIG Data Center (89), Beijing Institute of Genomics (BIG), Chinese Academy of Sciences, under accession numbers CRA001558 that are publicly accessible at http://bigd.big.ac.cn/gsa.

## Author Contributions

JL, YC, and JW conceived and designed the experiments. YG, LW, KL, ZZ, and MZ performed the experiments. LW, KL, JY, HY, YG, YH and JL analyzed the data. LW, KL, JY, HY, YG, JL, FY, JH, HY, and HMS contributed reagents, materials, and analysis tools. LW, KL, JY, HY, YG, JL, FY, JH, HY, and HM wrote the manuscript. All authors contributed to the article and approved the submitted version.

## Conflict of Interest

MZ was employed by the company Shanghai Suosheng Biotechnology Co., Ltd. The remaining authors declare that the research was conducted in the absence of any commercial or financial relationships that could be construed as a potential conflict of interest.

## Footnotes

1 https://github.com/jstjohn/seqprep

2 www.ingenuity.com

## Supplementary Material

Supplementary Table 1 Virus content.

Supplementary Table 2 Data quality control.

Supplementary Table 3 Correlation coefficient of samples.

Supplementary Table 4 Expression level of host differentially expressed genes.

Supplementary Table 5 GO enrichment of host differentially expressed genes.

Supplementary Table 6 KEGG enrichment of host differentially expressed genes.

Supplementary Table 7 KEGG energy metabolism of differentially expressed genes.

Supplementary Table 8 Sirtuin pathway of host differentially expressed genes.

Supplementary Table 9 The primer of quantitative real-time PCR.
